# Mr. Cooke, on Contagious Fever

**Published:** 1802-03

**Authors:** Charles Cooke

**Affiliations:** Glocester


### *To the Editors of the Medical and Physical Journal.*

GENTLEMEN,

THE Medical World is indebted to you for a Publication, which enables it to know every remedy propoſed, whether the ſame plan has been adopted by other practitioners, and with what advantage, and to judge of the effect by an accumulation of experiments; therefore, as you have inſerted in your valuable Miſcellany a Letter addreſſed by me to the Printer of the Glocefter Journal, ſigned A MEDICAL PRACTITIONER, I may be expected to explain my idea of the effect produced by waſhing with Vinegar.

I conceive that the effluvia (which I have, perhaps, incorrectly termed human atmoſphere) generated and thrown out upon the ſkin during the progreſs of Fever, has putrid and alkaline properties, which, when ſeparated from and ſurrounding the body of the patient, become one moſt material ſource of communicating the diſeaſe; conſequently, vinegar being antiſeptic and acid, when combined immediately (at the ſource) with this effluvia, produces an inert neuter, whereby the progreſs of Fever is checked from extending the contagious influence amongſt the attendants, and the type of fever, if not ſometimes altered, is meliorated in the ſymptoms and violence upon the patient.

By well rubbing after the vinegar is uſed, the ſkin is perfectly cleared from any remaining impurity, and a degree of perſpiration enſues, which is always comfortable to the patient. If indigent circumſtances will not afford the purchaſe of flannel dreſſes, I adviſe a renewal of blankets or ſheets, well aired, and the bed warmed every time after the vinegar is uſed.

I was induced to recommend the uſe of vinegar through the medium of the Glocefter Journal, for the benefit of our neighbouring poor; and by this practice amongſt them, I have experienced perienced every advantage; I find their habitations more free from filth; I think I have lefs reafon to dread contagion; and I obferve that thofe who were liable to the attack of the fame fever, (from being obliged to fleep in the fame bed, or in the fame room, with the fick) continue in health.

Is it not probable that, in times like the prefent, nothing would fo fpeedily exterminate or prevent fever in Poor-houfes, Houfes of Induftry, Prifons, &c. as a general wafhing of every individual once or twice in a week with ftrong vinegar?

Anxious to be ferviceable in that profeffion of which I am proud to call myfelf a member, I beg to fubfcribe myfelf,

Your's, &c. &c.

*Glocester, Jan.* 10, 1802. CHARLES COOKE.

P. S. The power of communicating the contagion of fever appears to be very limited, excepting by immediate contact; it does not feem, at fartheft, to extend beyond the fick-room; I do not think it very diffufive in the air. I have attended patients with fever in almoft every houfe in one parifh in this county, and the furrounding parifhes which adjoin have hardly an inftance of the fame difeafe. In another parifh, (on the oppofite fide of Glocefter) a woman was taken ill with fever; the eldeft daughter was very foon attacked in the fame manner; I then pronounced the fever contagious. From this time the neighbours would not go near the houfe. Three other children, with the woman's hufband, became ill fhortly afterward: The whole family, however, recovered; and I cannot learn that the fever extended itfelf farther in the parifh.

